# Matrix Metalloproteinase-9 −1562C/T Gene Polymorphism Is Associated with Diabetic Nephropathy

**DOI:** 10.1155/2016/1627143

**Published:** 2016-08-18

**Authors:** Shufen Feng, Gang Ye, Shi Bai, Hongcheng Wei, Xueling Liao, Lu Li

**Affiliations:** ^1^Department of Gastroenterology, The First Affiliated Hospital of Jinan University, Guangzhou 510630, China; ^2^Clinical Laboratory, People's Hospital of New District Longhua, Shenzhen 518109, China; ^3^Department of Nephrology, Affiliated Hospital of Guilin Medical College, Guangxi 541001, China

## Abstract

To investigate the association between the metalloproteinase-9 (MMP9) −1562C/T polymorphism and diabetic nephropathy (DN) in Han Chinese, the patients with type 2 diabetes were collected and divided into the non-DN (NDN) and DN groups; controls were recruited. Genotype and allele frequencies were assessed using polymerase chain reaction and restriction fragment length polymorphism. Results showed that SBP, DBP, HbA1c, UAER, Cr, BUN, TG, and TC were higher in the DN group compared with the control and NDN groups. SBP, HbA1c, and TC in DN patients with the TT and CT genotypes were lower than in those with CC. Compared with controls, the frequency of the T allele in the DN group was significantly lower. The MMP9 −1562C allele, SBP, Cr, BUN, TG, and TC were independent risk factors for DN. All of the above suggested that the MMP9 −1562C/T polymorphism was associated with DN in Han Chinese.

## 1. Introduction

Type 2 diabetes mellitus (T2DM) is a metabolic disorder characterized by hyperglycemia resulting from insulin resistance [1]. T2DM is a severe condition and is often associated with life-threatening conditions such as heart disease and stroke. The worldwide prevalence of T2DM is about 387 million. The incidence of T2DM is increasing worldwide, particularly in Asian countries. The prevalence of T2DM in China in 2014 was about 96.3 million [2]. Diabetic nephropathy (DN) occurs in almost 40% of patients with T2DM [3] and is one of the leading causes of T2DM-related death. DN is characterized by progressive kidney damage resulting from T2DM, but the risk factors for DN vary among populations. Therefore, a better understanding of the pathogenesis of DN is critical for early disease intervention.

DN development is related to inflammation and matrix metalloproteinases (MMP) play key roles in the progression of DN [4]. Several studies indicated that MMP9 is an important inflammatory marker involved in the pathophysiology of DN [5]. Proteins belonging to the MMP family are involved in the breakdown of extracellular matrix (ECM) in normal physiological processes such as tissue remodeling, but degradation of the ECM by MMP is also involved in the pathogenesis of a number of diseases [6]. Major pathological changes are associated with DN and result from the accumulation of ECM in the glomerular mesangium and the renal interstitium, leading to progressive glomerulosclerosis and renal interstitial fibrosis [7]. MMP9 plays a critical role in diabetic complications such as diabetic retinopathy and DN [8]. In addition, MMP9 is detectable in a number of other renal conditions such as immunoglobulin A nephropathy, membranoproliferative glomerulonephritis, and lupus nephritis [9]. Bernerine has renal protective effects against DN through the regulation of MMPs [10]. Therefore, taken together, there might be an association between MMP9 and DN, but whether MMP9 promotes or inhibits the development of DN is still controversial [11].

Several mechanisms may be responsible for regulating MMP9 expression and activity in DN, and single nucleotide polymorphisms (SNPs) within the MMP9 gene might influence its expression. The effects of a number of SNPs have been described for a number of MMPs [12]. A recent paper reported SNP in the MMP9 gene promoter that reduces the risk of diabetic microvascular complications [13], but there is no study specifically examining MMP9 SNPs in DN. A previous study showed that the MMP9 −1562C/T SNP was associated with diabetic macroangiopathy [14], but there is no study specifically examining MMP9 SNPs in DN.

Therefore, the present study examined SNPs in the MMP9 gene among Han Chinese individuals with DN, without DN (NDN), and without kidney disease (controls).

## 2. Materials and Methods

### 2.1. Subjects

This case-control study included 310 patients diagnosed with T2DM at the Endocrinology Department of The First Affiliated Hospital of Jinan University (Guangzhou, China) between January 2013 and August 2013. Diagnosis of T2DM was in line with the diagnostic and classification criteria for diabetes established by the World Health Organization (WHO) in 1999 [15]. Patients with T2DM were categorized into the DN group (*n* = 168; 78 males and 90 females) and the NDN group (*n* = 142; 80 males and 62 females). Diagnostic criteria for DN included urinary albumin excretion rate (UAER) >30 mg/24 h, serum creatinine (Cr) >167 *μ*mol/L, and blood urea nitrogen (BUN) >7.8 mmol/L, confirmed in at least two independent consecutive examinations [16]. Patients with other primary/secondary renal diseases or those who had used nephrotoxic drugs were excluded.

During the same period, 100 healthy volunteers were recruited as controls (55 males and 45 females). Individuals with cardiovascular diseases, a history of diabetes, or a family history of liver, kidney, endocrine, or metabolic disease were excluded.

All subjects were unrelated to one another. The present study was approved by the Ethics Committee of The First Affiliated Hospital of Jinan University. All subjects agreed to have blood drawn and provided signed informed consent.

### 2.2. Clinical Data

Whole blood (5 mL) was sampled from each fasting participant in EDTA-containing tubes; 2 mL was stored at −20°C for DNA extraction and 3 mL was used for plasma separation and detection of fasting glucose (FPG), Cr, BUN, triglycerides (TG), total cholesterol (TC), and other biochemical markers using an automatic biochemical analyzer. Glycated hemoglobin (HbA1c) was detected using an HbA1c analyzer. UAER was detected using a radioimmunoassay. Systolic blood pressure (SBP) and diastolic blood pressure (DBP) were measured after a 5 min rest in the sitting position.

### 2.3. Polymorphism Analysis

Genomic DNA was extracted from whole blood using a blood genomic DNA extraction kit (Shanghai Generay Biological Engineering Co., Ltd., Shanghai, China) and stored at −20°C. PCR primers were designed and synthesized by Shanghai Sangon Biological Engineering Technology Services Ltd. (Shanghai, China). The primers for the MMP9 promoter region were as follows: sense 5′-CTT CCT AGC CAG CCG GCA TC-3′ and antisense 5′-GCC TGG CCT ATA GTA GGC CC-3′. The target sequence was amplified by PCR, generating an amplification product of 435 bp. The PCR reaction volume was 25 *μ*L and included 0.5 *μ*L of each primer, 1.5 *μ*L of template DNA, 12.5 *μ*L of TaqDNA polymerase (1.25 U), and sterile double-distilled water. The reaction conditions were as follows: (1) 94°C for 5 min; (2) 35 cycles of amplification at 94°C for 35 s, 60°C for 30 s, and 72°C for 45 s; and (3) 72°C for 5 min. PCR was performed using an Applied Biosystems PCR System. PCR amplification product (3 *μ*L) was digested with 2 U of the* SphI* restriction endonuclease at 37°C for 16 h to detect the MMP9 −1562C/T SNP. After digestion, the products were separated on 3% ethidium bromide-stained agarose gels (Biowest, Madrid, Spain). Images were acquired and analyzed using a gel imaging system.

### 2.4. Statistical Analysis

Data analysis was performed using SPSS 18.0 (IBM, Armonk, NY, USA). The Hardy-Weinberg equilibrium was used to verify that all samples were representative of the general population. Continuous data were expressed as mean ± standard deviation. Categorical data were expressed as frequencies. Intergroup comparisons were performed using analysis of variance (ANOVA) and the Bonferroni post hoc test. Genotype and allele frequencies were compared among groups with the chi-square test. Logistic regression analysis was conducted to explore the relationship between genotypes and relative risk of DN and was expressed as odds ratio (OR) and 95% confidence intervals (CI). Two-sided *P* values < 0.05 were considered statistically significant.

A post hoc power analysis was performed using the QUANTO software (http://biostats.usc.edu/software). Considering that the prevalence of DN among diabetic Chinese subjects is about 20% (*K*_*p*_) and considering that the frequency of the null/null genotype among study subjects is 53%, *Q*_*A*_ = √0.53 = 0.728. The hazard ratio of the null/null genotype patients compared with healthy subjects is therefore 2.27 (*R*_*g*_). Consequently, using a power of 0.8 and two-sided *α* of 0.05, the minimal sample size was 99.

## 3. Results

### 3.1. Characteristics of the Subjects

There was no statistical difference in gender, age, height, or BMI (all *P* > 0.05) among the three groups. SBP, DBP, HbA1c, UAER, Cr, BUN, TG, and TC were all significantly higher in the DN group compared with the control group (all *P* < 0.05). SBP, DBP, HbA1c, Cr, BUN, TG, and TC were also significantly higher in the NDN group compared with the control group (all *P* < 0.05). Finally, SBP, HbA1c, UAER, Cr, BUN, TG, and TC were all significantly higher in the DN group compared with the NDN group (all *P* < 0.05) (Table 1).

### 3.2. MMP9 −1562C/T Allele SNPs

**Figure 1:**
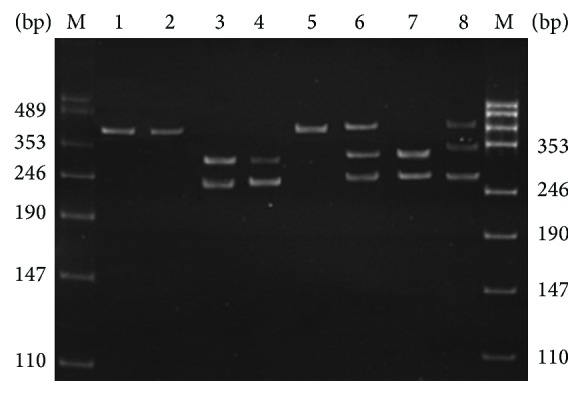
Electrophoresis of amplification fragments of the MMP9 promoter region for the detection of the −1562C/T polymorphism. Lanes 1, 2, and 5 are the CC genotype; lanes 3, 4, and 7 are the TT genotype; lanes 6 and 8 are the CT genotype. M: DNA molecular weight marker.

The CC genotype was represented by a single 435-bp band. The TT genotype was identified by two bands (247 and 188 bp). Finally, the CT genotype was represented by three bands (435, 247, and 188 bp (Figure 1)).

### 3.3. Genotype Distribution and Allele Frequencies of the MMP9 −1562C/T Allele

The Hardy-Weinberg equilibrium test showed that the distribution of the MMP9 −1562C/T allele in the NC, NDN, and DN groups was in line with the law of genetic equilibrium (*P* > 0.05) (Table 2). There were significant differences in allele and genotype frequencies between the DN and NC groups (*P* < 0.05). The difference in genotype between the DN and NC groups had a recessive inheritance pattern (CC versus CT + TT) and was statistically significant (*P* < 0.001). The frequency of the T allele in the DN group was significantly lower than in the NC group (*P* = 0.043). The relative risk of suffering from DN in subjects carrying the T allele was 0.478 compared with individuals with the C allele (OR = 0.478, 95% CI: 0.288–0.791). There was no difference in allele and genotype frequencies between the NC and NDN groups (*P* > 0.05). The genotype frequencies of the DN group were different compared with the NDN group (*P* < 0.05), but there was no significant difference in allele frequency (*P* > 0.05) (Table 3).

### 3.4. Comparison of Blood Glucose, Blood Pressure, and Blood Lipids in Patients with DN

SBP, HbA1c, and TC levels in patients with DN and the MMP9 −1562 TT and CT genotypes were significantly lower than in those with the CC genotype (all *P* < 0.05) (Table 4).

### 3.5. Multivariate Analysis

To predict the probability of DN in the NDN and DN groups, MMP9 −1562C genotypes (dummy variables were created and the TT genotype was used as control), DBP, SBP, HbA1c, Cr, BUN, TG, TC, and UAER were entered as independent variables, and the binary logistic regression analysis was performed using the forward selection procedure. MMP9 −1562C genotypes (CT: OR = 1.531, 95% CI: 1.186–1.975, *P* = 0.001; CC: OR = 7.402, 95% CI: 4.534–12.083, *P* < 0.001), SBP (OR = 1.6, 95% CI: 1.243–2.081, *P* < 0.001), Cr (OR = 6.798, 95% CI: 4.357–12.479, *P* = 0.042), BUN (OR = 4.423, 95% CI: 2.765–9.831, *P* = 0.006), TG (OR = 6.466, 95% CI: 3.047–11.926, *P* = 0.039), and TC (OR = 3.522, 95% CI: 2.105–6.269, *P* = 0.001) were independent risk factors for DN (Table 5).

## 4. Discussion

In this study, the relationship between MMP9 SNPs and DN was examined in the Han Chinese population in Guangzhou (China). SBP, DBP, HbA1c, UAER, Cr, BUN, TG, and TC were higher in the DN group compared with the control and NDN groups. SBP, HbA1c, and TC in DN patients with the TT and CT genotypes were lower than in those with the CC genotype. The relative risk of DN in individuals carrying the T allele was 0.478 compared with individuals carrying the C allele. The MMP9 −1562C genotype, SBP, Cr, BUN, TG, and TC were independent risk factors for DN.

The MMP9 gene is located on the chromosome 20q11.2–13.1 in humans and contains four promoter regions. MMP9 −1562C/T is located upstream of the promoter and contains binding sites for transcriptional repressors [17, 18]. In the presence of the T allele, transcriptional repression is decreased or eliminated, probably leading to elevated expression of MMP9, increased degradation of ECM, and delayed interstitial fibrosis and renal glomerular sclerosis; as a result, DN development and progression are delayed. Therefore, the C allele probably alters MMP9 expression to promote the occurrence and development of DN. A previous study has shown that the T allele of the MMP9 −1562C/T SNP was associated with diabetic macroangiopathy [14]. Two studies showed that the T allele was associated with diabetic foot ulcers and nonhealing wounds in diabetic subjects [19, 20]. This discrepancy could be related to a number of factors, including the subjects themselves, the choice of the controls, and the nature and pathogenesis of the lesions (macroangiopathy versus DN). Additional studies are necessary to solve this issue.

Compared with the control and NDN groups, SBP, HbA1c, UAER, Cr, BUN, TG, and TC were all elevated in the DN group, suggesting that blood pressure, blood glucose level, and kidney function are important factors involved in DN. SBP, HbA1c, and TC levels in patients with DN and the TT/CT genotypes were significantly lower than in patients with the CC genotype. In addition, the recessive inheritance pattern (i.e., CC versus CT + TT) between the DN and control groups was statistically different. Compared with the control group, the T allele frequency in the DN group was significantly lower, and the relative risk of DN in individuals carrying the T allele was only 0.478 compared with the individuals carrying the C allele. In the present study, the MMP9 −1562C genotype, SBP, Cr, BUN, TG, and TC were all independent risk factors for the occurrence of DN. These findings strongly suggest that the T allele is probably a protective factor against the development of DN, while the C allele probably promotes DN development.

A number of groups have previously performed genome-wide association studies to identify genetic variants associated with the risk of developing T2DM [21–23]. To date, there are approximately 70 loci that are significantly associated with T2DM, but it is important to note that many of these SNPs are in noncoding regions. Thus, the exact mechanisms by which they influence the risk of disease are not very well understood. Along these same lines, many studies have identified genomic regions associated with susceptibility to DN, but the identification of a single causal variant has not been reported. The present study is unique in that the −1562C/T variant occurs within the MMP9 promoter and within a region that is known to influence the expression of MMP9. This provides a mechanistic basis explaining why the C allele contributes to DN and why the T allele is protective. It would be interesting to explore if the −1562C/T variant identified in this study adds to the predictive power of any of the previously identified T2DM susceptibility alleles, but additional comprehensive and in-depth studies are necessary to address this issue.

This study is not without limitations. The sample size was relatively small and from a single geographic region. Indeed, there are differences in genotypes and allele frequencies between different ethnic groups and/or regions [24]. Additional studies are necessary to examine the contribution of the MMP9 SNPs to DN.

## 5. Conclusions

The MMP9 −1562C/T SNP was associated with DN in a Han Chinese population. The T allele was a protective factor for DN, while the C allele contributed to the disease.

## Figures and Tables

**Table 1:** Characteristics of the subjects.

	NC	NDN	DN
Cases	100	142	168
Gender (M/F)	55/45	80/62	78/90
Age (years)	57.07 ± 10.82	56.48 ± 9.10	54.67 ± 11.81
Height (cm)	164.52 ± 6.53	165.37 ± 7.12	166.17 ± 7.08
BMI (kg/m^2^)	21.47 ± 1.08	24.58 ± 2.07	25.09 ± 1.92
SBP (mmHg)	121.68 ± 7.13	138.41 ± 8.43^*∗∗*^	149.51 ± 8.65^##*∗∗*^
DBP (mmHg)	84.78 ± 9.41	106.83 ± 10.26^*∗∗*^	111.42 ± 9.30^*∗∗*^
HbA1c (%)	4.98 ± 0.85	6.51 ± 0.81^*∗∗*^	8.01 ± 0.96^##*∗∗*^
UAER (mg/24 h)	17.14 ± 2.51	20.78 ± 1.59	301.93 ± 51.32^##*∗∗*^
Cr (*μ*mol/L)	60.51 ± 7.21	97.92 ± 15.23^*∗∗*^	129.71 ± 23.53^##*∗∗*^
BUN (mmol/L)	4.04 ± 0.51	5.47 ± 0.67^*∗∗*^	9.43 ± 0.82^##*∗∗*^
TG (mmol/L)	1.06 ± 0.08	2.13 ± 0.24^*∗∗*^	2.25 ± 0.32^##*∗∗*^
TC (mmol/L)	4.12 ± 0.26	4.58 ± 0.49^*∗∗*^	4.96 ± 0.54^##*∗∗*^

^*∗∗*^ *P* < 0.01 versus the NC group.

^##^ *P* < 0.01 versus the NDN group.

BMI: body mass index; SBP: systolic blood pressure; DBP: diastolic blood pressure; UAER: urinary albumin excretion rate; TC: total cholesterol; TG: triglycerides; Cr: creatinine; BUN: blood urea nitrogen.

**Table 2:** Genetic equilibrium test for MMP9 gene −1562C/T mutation.

		NC	NDN	DN
Cases		100	142	168

Genotype frequency	CC	45/48.05	64/65.86	101/98.52
	CT	13/11.25	30/31.26	37/35.01
	TT	42/40.70	48/44.74	30/34.47

*P*		0.79	0.86	0.62

**Table 3:** Distribution frequency of MMP9 −1562C/T alleles [case (%)].

		NC	NDN	DN
*N*		100	142	168

Genotype frequency	CC	45 (45.0)	64 (45.1)	101 (60.1)
	CT	13 (13.0)	30 (21.1)	37 (22.0)^#^
	TT	42 (42.0)	48 (33.8)	30 (17.9)^*∗∗*^

Allele frequency	C alleles	53 (53.0)	75 (52.8)	119 (52.8)
	T alleles	47 (47.0)	67 (47.2)	49 (29.2)^*∗∗*^

^*∗∗*^ *P* < 0.01 versus the NC group.

^#^ *P* < 0.05 versus the NDN group.

**Table 4:** Comparison of blood pressure, blood glucose, and blood lipid levels between the DN patients carrying MMP9 −1562T and MMP9 −1562C genotypes.

	CT + TT	CC	*P*
Cases	67	101	
SBP (mmHg)	138.41 ± 8.43	149.51 ± 8.65	0.032
DBP (mmHg)	106.83 ± 10.26	111.42 ± 9.30	0.612
HbA1c (%)	6.51 ± 0.81	8.01 ± 0.96	0.004
TC (mmol/L)	4.79 ± 1.08	5.48 ± 1.25	0.017
TG (mmol/L)	1.85 ± 0.84	1.86 ± 0.91	0.057

SBP: systolic blood pressure; DBP: diastolic blood pressure; TC: total cholesterol; TG: triglycerides.

**Table 5:** Multivariate logistic regression analysis.

Variables		OR	95% CI	*P*
MMP9 −1562C	TT	Reference	—	—
	CT	1.531	1.186–1.975	0.001
	CC	7.402	4.534–12.083	<0.001

SBP		1.600	1.243–2.081	<0.001

TC		3.522	2.105–6.269	0.001

TG		6.466	3.047–11.926	0.039

Cr		6.798	4.357–12.479	0.042

BUN		4.423	2.765–9.831	0.006

OR: odds ratio; 95% CI: 95% confidence interval; SBP: systolic blood pressure; TC: total cholesterol; TG: triglycerides; Cr: creatinine; BUN: blood urea nitrogen.
